# *Polyopes affinis* Suppressed IFN-γ- and TNF-α-Induced Inflammation in Human Keratinocytes via Down-Regulation of the NF-κB and STAT1 Pathways

**DOI:** 10.3390/molecules27061836

**Published:** 2022-03-11

**Authors:** Yuna Ha, Won-Hwi Lee, Jang Kyun Kim, Hee-Kyung Jeon, Jongsung Lee, Youn-Jung Kim

**Affiliations:** 1 Research Institute of Basic Sciences, Incheon National University, Incheon 22012, Korea; dbsk335@hanmail.net (Y.H.); dnjsgnl_@naver.com (W.-H.L.); jang.kim@inu.ac.kr (J.K.K.); 2 Department of Marine Sciences, Incheon National University, Incheon 22012, Korea; 3 Advanced Energy Materials and Components R&D Group, Korea Institute of Industrial Technology, Busan 46938, Korea; jeonhk75@kitech.re.kr; 4 Department of Integrative Biotechnology, College of Biotechnology and Bioengineering, Sungkyunkwan University, Suwon City 16419, Gyunggi Do, Korea

**Keywords:** *Polyopes affinis*, atopic dermatitis, TARC/CCL17, MDC/CCL22, MAPKs, STAT1, NF-κB

## Abstract

*Polyopes affinis* is a red algal species commonly found on the South coast and near Jeju Island, Korea. This study aimed to determine whether *P. affinis* extracts can inhibit the pathogenesis of T-helper-2 (Th2)-mediated inflammation in a human keratinocyte cell line of atopic dermatitis (AD). Cells were incubated with 10 ng/mL of interferon gamma (IFN-γ) and 10 ng/mL of tumor necrosis factor-alpha (TNF-α) at various concentrations of PAB (10, 30, and 60 µg/mL) and PAA (100, 500, and 1000 µg/mL) extracts. A gene-ontology (GO)-enrichment analysis revealed that PAB significantly enriched the genes associated with biological processes such as cell adhesion, immune response, inflammation, and chemokine-mediated pathways. PAB suppressed the expression of the secretory proteins and mRNAs that are associated with the thymus and the production of activation-regulated chemokines (TARC/CCL17) and macrophage-derived chemokines (MDC/CCL22). The effect of the extract on mitogen-activated protein kinases (MAPKs) was related to its inhibition of TARC/CCL17 and MDC/CCL22 production by blocking NF-κB and STAT1 activation. These results suggest that seaweed extract may improve AD by regulating pro-inflammatory chemokines. In conclusion, we first confirmed the existence of phloroglucinol, a polyphenol formed from a precursor called phlorotannin, which is present in PAB, and this result proved the possibility of PAB being used as a treatment for AD.

## 1. Introduction

Except for industrial food processing, seaweed is not widely consumed in Western countries (with the exception of Scotland and Ireland), but is widely consumed in some East Asian countries such as Korea, China and Japan [1]. Seaweed is one of the representative edible marine resources with relatively little research as a wellness material and high potential compared to land plants. Because of the beneficial effect of marine seaweed, its consumption has increased yearly, reaching a mean of 5 kg a year for an ordinary Korean adult [2]. Therefore, it was thought to be necessary to evaluate the anti-inflammatory effect of seaweed, which grows naturally along the domestic coast and is one of the marine resources that have materials with abundant potential. The constituents of seaweeds can be largely divided into pigment substances, amino acids, sterols, and polyphenols. As a well-known example, fucoxanthin, a rich pigment component of brown algae, has been reported to have anti-obesity, anti-bacterial, and whitening effects [3,4,5,6]. In addition, according to a recent study, it has been reported that the phlorotannins, which are a kind of polyphenol, as well as a carotenoid-based pigment component and a flavonoid component, have anti-inflammatory effects [7,8,9,10]. *Polyopes affinis* is one of the representative edible red algae and grows mainly on intertidal rocks, and in Korea it mainly lives on the southern coast or Jeju Island. However, there are no studies on the efficacy of *P. affinis* in atopic dermatitis (AD). Therefore, in this study, we evaluated the effect on AD of the red algae *P. affinis*, which is mainly found on the southern coast of Korea and is also used in food.

AD is one of the most common chronic skin diseases, and its causes have not been identified [11,12,13,14]. Although there is no clear cause of the disease, it is characterized by skin-barrier abnormalities, environmental factors, imbalanced immune responses, and genetic factors. AD is accompanied by itching, flushing, and dysfunction of the skin barrier, which can lead to bacterial penetration and allergic reactions, which can in turn lead to swelling. From an immunological point of view, characteristics of AD have been shown to be related to the abnormal overexpression and modulation of the thymus and the secretion of activation-regulated chemokines (TARC/CCL17) and macrophage-derived chemokines (MDC/CCL22) in human keratinocytes [15].

One of the most important aspects of AD is that it is characterized by the predominant infiltration of inflammatory cells such as macrophages, mast cells, eosinophils, and other cells, and an increased secretion of Th2-related response factors by the production of tumor necrosis factor-α (TNF-α) and interferon-γ (IFN-γ) [16,17]. The relationship between AD and keratinocytes is that keratinocytes play a pivotal role in the development of inflammatory skin diseases. Keratinocyte cells, which exist in more than 80% of the human stratum corneum, recognize them as antigens when stimulated from outside the skin. At this time, antigen-presenting cells promote the production of secretion proteins such as cytokines and chemokines through interaction with helper T cells to transmit signals involved in the immune response. The cells produce different chemokines and pro-inflammatory cytokines, especially TARC/CCL17 and MDC/CCL22, which regulate the activation of normal T-cell, secretory-protein, and intercellular-adhesion-molecule-1 (ICAM-1) expression in response to stimulation by TNF-α/IFN-γ.

Therefore, the main purpose of this study was to evaluate whether the *P. affinis* extract, which has not been studied thus far, can be used as a therapeutic natural substance for AD. To confirm the mRNA expression levels of TARC/CCL17 and MDC/CCL22, which are known AD biomarkers, real-time PCR was performed by treating the HaCaT cell line that was stimulated with IFN-γ and TNF-α with the *P. affinis* extract. In addition, the level of secreted proteins was measured by ELISA, and significant pathway modulators present in *P. affinis* were identified. In this study, we examined the effects of *P. affinis* on activated, spontaneously immortalized human keratinocytes, an “AD-like” HaCaT cell line induced by IFN-γ and TNF-α, to identify new potential targets for therapy and/or to develop a tool for AD treatment.

## 2. Results

### 2.1. Cytotoxic Effects of PAA and PAB in HaCaT Cells

**Figure 1:**
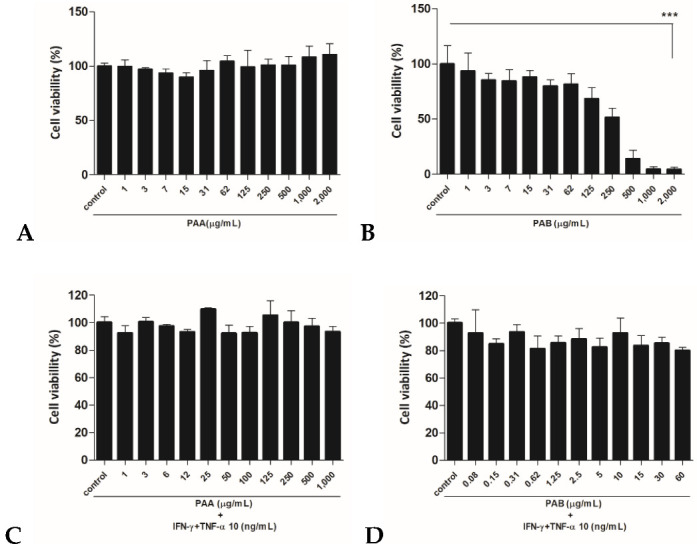
Cell viability was determined using the MTT assay. Cells were seeded onto 96-well plates and treated with various concentrations of PAA (control, 1, 3, 7, 15, 31, 62.5, 125, 250, 500, 1000, or 2000 μg/mL) and PAB (control, 1, 3, 7, 15, 31, 62.5, 125, 250, 500, 1000, or 2000 μg/mL) for 24 h (**A**,**B**). Cells were incubated with indicated concentration of cytokines (IFN-γ and TNF-α) combined with PAA or PAB for 24 h (**C**,**D**). The values are expressed as the mean ± standard deviation (SD) of three independent experiments. Note: *** *p* < 0.001 versus control cells.

To evaluate the toxicity of seaweed extracts, its cytotoxicity in HaCaT cells was measured using the MTT assay (Figure 1). In the water fraction (PAA), no significant cytotoxicity was observed up to the highest concentration of 2000 μg/mL. However, in the butanol-fraction layer (PAB), cell viability decreased in a dose-dependent manner. PAB was found to be more toxic than PAA (Figure 1A,B). The concentration at which more than 80% of the cells survived was selected as the highest concentration, and it was treated with IFN-γ and TNF-α. When 1000 μg/mL for PAA and 60 μg/mL for PAB were selected as the highest concentration and treated with IFN-γ and TNF-α, significant cytotoxicity was not observed (Figure 1C,D).

### 2.2. Effects of PAA and PAB on IFN-γ- and TNF-α-stimulated TARC/CCL17 and MDC/CCL22mRNA Expression in HaCaT Cells

**Figure 2:**
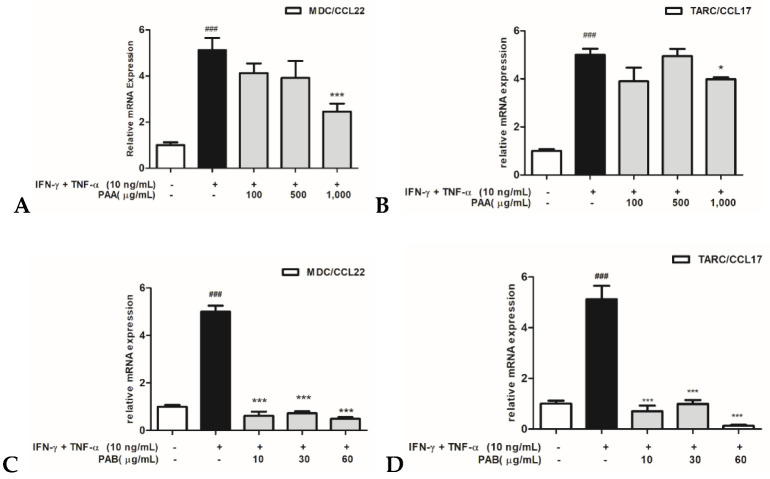
PAA and PAB inhibit mRNA expression of inflammatory chemokines in IFN-γ + TNF-α-stimulated HaCaT cells. mRNA expression of TARC/CCL17 (**A**,**C**) and MDC/CCL22 (**B**,**D**) were measured using the RNA extracted from cells treated with PAA (100, 500, and 1000 μg/mL) or PAB (10, 30, and 60 μg/mL) and IFN-γ and TNF-α (10 ng/mL each) for 24 h. After treatment, mRNA expression was measured and normalized to GAPDH expression. IFN-γ and TNF-α columns induced were represented by black bars, and various PAA or PAB concentrations columns were represented by gray bars. Values are expressed as the mean ± SEM of three independent experiments. Note: ^#^ *p*< 0.05, ^##^ *p*< 0.01, and ^###^ *p*< 0.001 versus control cells; * *p* < 0.05, ** *p* < 0. 01, and *** *p* < 0.001 versus IFN-γ- and TNF-α-induced cells.

Real-time PCR was performed to assess the gene-expression patterns of pro-inflammatory chemokines in HaCaT cells that were induced by IFN-γ and TNF-α (Figure 2). The treatment of IFN-γ and TNF-α in TNF-α-induced HaCaT cells significantly induced TARC/CCL17 and MDC/CCL22. Conversely, the highest concentration (1000 μg/mL) of PAA showed a relatively inhibitory effect on TARC/CCL17 and MDC/CCL22. Similarly, the PAB-treated HaCaT cells showed a significant inhibition of TARC/CCL17 and MDC/CCL22 in a dose-dependent manner compared to the IFN-γ- and TNF-α-induced group. PAB inhibited the mRNA expression of TARC/CCL17 and MDC/CCL22 more significantly than PAA.

### 2.3. Effects of PAA and PAB on IFN-γ- and TNF-α-Stimulated TARC/CCL17 and MDC/CCL22 Secretory Proteins in HaCaT Cells

**Figure 3:**
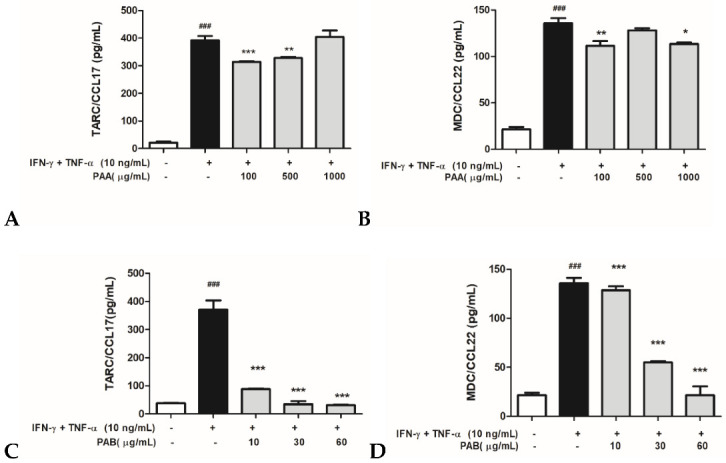
Cell supernatants were prepared from the HaCaT cells treated with IFN-γ and TNF-α, and the levels of TARC/CCL17 (**A**,**C**) and MDC/CCL22 (**B**,**D**) production were determined using ELISA. The cells were co-treated with various concentrations of PAA (100, 500, and 1000 μg/mL) or PAB (10, 30, and 60 μg/mL) and stimulated with IFN-γ and TNF-α (10 ng/mL each) for 24 h. IFN-γ and TNF-α columns induced were represented by black bars, and various PAA or PAB concentrations columns were represented by gray bars. Values are expressed as the mean ± standard deviation (SD) of three independent experiments. Note: ^#^ *p*< 0.05, ^##^ *p*< 0.01, and ^###^ *p*< 0.001 versus control cells; * *p* < 0.05, ** *p* < 0.01, and *** *p* < 0.001 versus TNF-α- and IFN-γ-induced cells.

To investigate the production of pro-inflammatory chemokines in the IFN-γ-, TNF-α/IFN-γ-, and TNF-α-induced HaCaT cells treated with PAA or PAB, the TARC/CCL17 and MDC/CCL22 levels were measured using ELISA. In the PAA-treated cells, the production of TARC/CCL17 was detected after the treatment with IFN-γ and TNF-α/IFN-γ and TNF-α at approximately 400 pg/mL compared to the control group (Figure 3A). Similarly, the MDC/CCL22-secreted protein was detected after treatment with approximately 140 pg/mL of IFN-γ and TNF-α (Figure 3B). Despite the increase in the levels of secreted protein, a slight inhibitory effect was observed in several of the treatment groups exposed to PAA. The exposure of cells that were induced with IFN-γ and TNF-α to PAB showed a marked concentration-dependent decrease in TARC/CCL17 and MDC/CCL22 levels (Figure 3C,D). Therefore, these results suggest that PAB down-regulates the production of TARC/CCL17 and MDC/CCL22 chemokines to regulate the inflammatory mechanism in IFN-γ- and TNF-α-stimulated HaCaT cells.

### 2.4. Effect of PAB on the Gene Expression Profile of IFN-γ- and TNF-α-Stimulated HaCaT Cells

**Figure 4:**
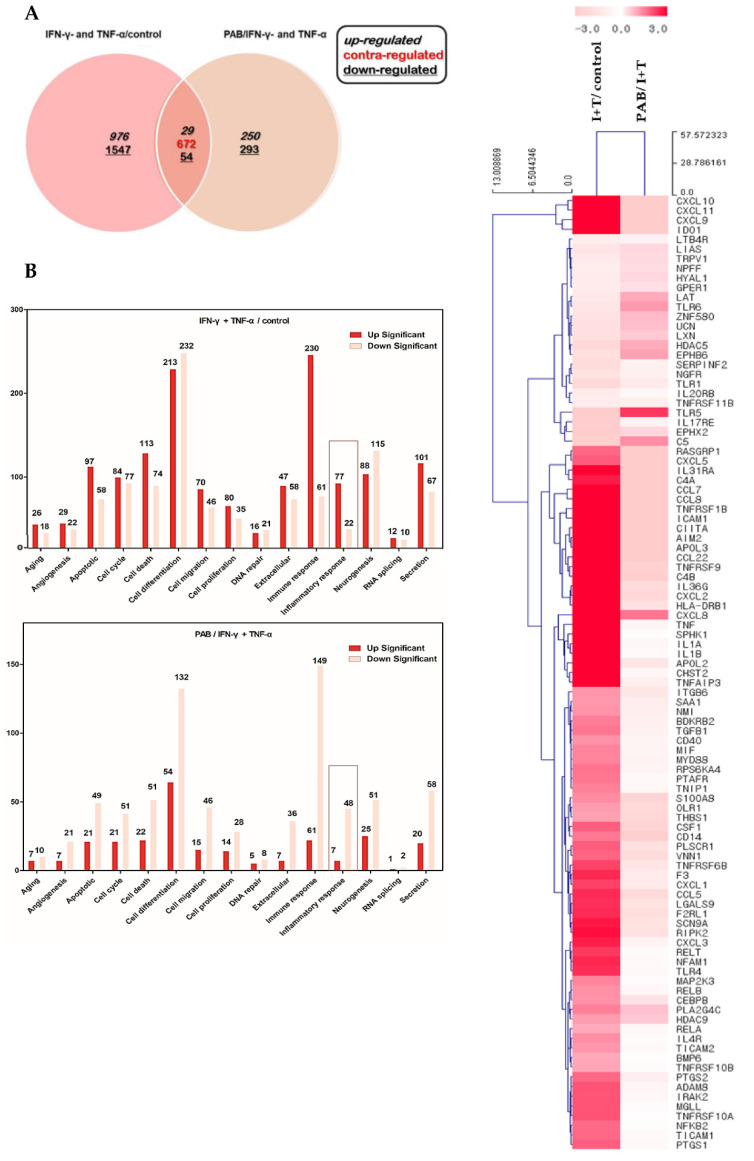
Overview of differentially expressed genes under IFN-γ and TNF-α stimulation and PAB treatment. (**A**) Venn diagram depicting the number of genes differentially expressed by IFN-γ and TNF-α compared to control and PAB-treated cells compared to IFN-γ- and TNF-α-treated HaCaT cells; (italic number) up-regulated genes, (underlined number) down-regulated genes, and (red number) counteract-regulated genes represent the number of DEGs (fold change > 2 and *p* < 0.05). (**B**) Gene-ontology (GO) category was analyzed using DAVID gene-functional-classification tool (https://david.ncifcrf.gov/, accessed on 18 January 2022). Percentages and number of genes classified into 15 GO categories showed expressional changes (up- or down-regulated). (**C**) Hierarchical clustering showed changes in the gene expression of IFN-γ- and TNF-α-induced cells compared to control HaCaT cells and PAB-treated cells compared to IFN-γ- and TNF-α-treated HaCaT cells. Fold-change of ≥ 2.0 and *p* < 0.05, compared to the vehicle control group (DMSO; pink: up-regulation; white: down-regulation).

To evaluate the effect of IFN-γ and TNF-α stimulation and PAB treatment on the gene-expression profile of the HaCaT cells, the gene-expression changes in the control, IFN-γ and TNF-α-stimulated cells, as well as the PAB co-treated conditions in the IFN-γ and TNF-α-stimulated HaCaT cells were analyzed using the statistical criterion *p* < 0.05. Only genes that displayed ≥ two-fold up-regulation or down-regulation were considered in this study (Figure 4A). In the IFN-γ- and TNF-α-stimulated group, the expression of 3278 genes (976 up-regulated and 1547 down-regulated) was changed; in the IFN-γ- and TNF-α-stimulated HaCaT cells of the PAB co-treated group, the expression of 1298 genes (250 up-regulated and 293 down-regulated) was changed. We found that 83 genes (29 up-regulated and 54 down-regulated) were commonly expressed in these two condition groups. The 672 genes in red were marked as contra-regulated. Here, the definition of a contra-regulated gene is an average gene that increases by IFN-γ and TNF-α treatment and decreases by PAB compared to the control, i.e., showing the opposite trend. To identify the functional changes in DEGs, a gene-ontology (GO) analysis was performed using ExDEGA software (E-Biogen Inc. Seoul, Korea) [18,19,20]. In this study, the GO categories were divided into 15 functional groups (Figure 4B). We performed a hierarchical clustering analysis using the gene expression of IFN-γ- and TNF-α-induced cells compared to the control HaCaT cells, and PAB-treated cells compared to IFN-γ- and TNF-α-treated HaCaT cells (Figure 4C). The genes of the 15 GO categories were up-regulated in the IFN-γ- and TNF-α-treated group compared to the control group, and after PAB treatment, compared to the IFN-γ- and TNF-α-induced treatment group, down-regulation of the genes of the 15 GO categories was observed. Among the 15 GO categories, additional analysis was performed that focused on the inflammatory response.

### 2.5. Nine Classified Genes and Interconnected Networks with TARC/CCL17 and MDC/CCL22

**Figure 5:**
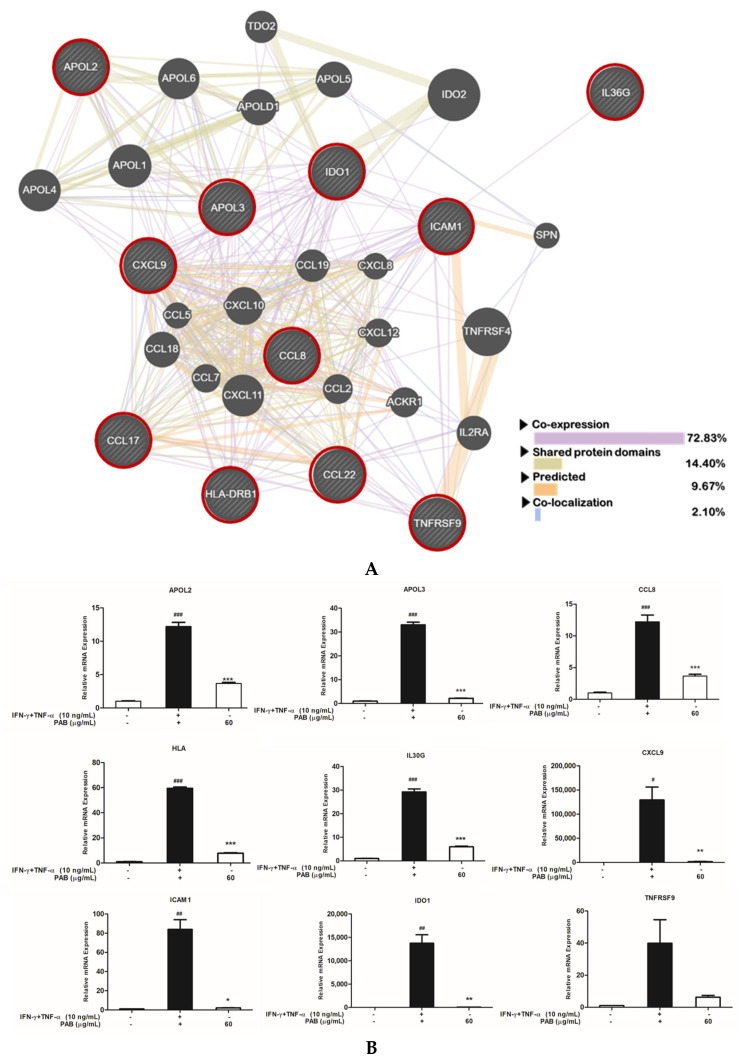
(**A**) Interaction analysis between the nine selected genes and CCL17 (TARC/CCL17) and CCL22 (MDC/CCL22). (**B**) TNFRSF9, CCL8, ICAM1, IL36G APOL3, APOL2, CXCL9, and HLA-DRB1 mRNA-expression values were measured and normalized to GAPDH expression. Values are expressed as the mean ± SEM of three independent experiments. IFN-γ and TNF-α columns induced were represented by black bars, and PAB 60 μg/mL treated columns were represented by white bars. Note: ^#^ *p*< 0.05, ^##^ *p*< 0.01, and ^###^ *p* < 0.001 versus control cells; * *p* < 0.05, ** *p* < 0. 01, and *** *p* < 0.001 versus TNF-α- and IFN-γ-induced cells.

In particular, nine chemokines thought to be of primary importance in skin inflammation were further analyzed. IFN-γ- and TNF-α-stimulated keratinocytes showed a selective up-regulation of the following proteins: tumor necrosis factor receptor superfamily member 9 (TNFRSF9), C-C motif chemokine ligand 8 (CCL8), intercellular adhesion molecule 1 (ICAM1), interleukin 36 gamma (IL36G), apolipoprotein L3 (APOL3), apolipoprotein L2 (APOL2), C-X-C motif chemokine ligand 9 (CXCL9), major histocompatibility complex class II, DR beta 1 (HLA-DRB1), and indoleamine 2,3-dioxygenase 1 (IDO1), whereas PAB was able to effectively inhibit the expression of the up-regulated genes (Table 1). As shown in Figure 5A, a total of nine genes, TNFRSF9, CCL8, ICAM1, IL36G APOL3, APOL2, CXCL9, HLA-DRB1, and IDO1, as well as the genes for the classical biomarkers TARC/CCL17 and MDC/CCL22, were analyzed for interaction using GeneMANIA. The CCL17 (TARC/CCL17) and CCL22 (MDC/CCL22) genes and the nine selected genes were analyzed, and their associations were subdivided into four categories: co-expression, shared protein domains, predicted, and co-localization, and a mutually networked analysis was performed. The results of the RNA sequencing and prediction analysis of genes associated with inflammatory responses were further validated using real-time RT-PCR. The RT-PCR-analysis results were consistent with the fold-change values (Figure 5B). Thus, PAB inhibited inflammation-mediating genes such as TNFRSF9, CCL8, ICAM1, IL36G APOL3, APOL2, CXCL9, HLA-DRB1, and IDO1. In addition, the interaction of the nine genes with TARC/CCL17 and MDC/CCL22 were analyzed, suggesting the possibility that these nine genes could also be utilized as inflammation-mediated biomarkers of AD.

### 2.6. Effect of PAB Regulation on Inflammation Related MAPKs and STAT1 Signaling Pathways in IFN-γ and TNF-α-Induced HaCaT Cells

**Figure 6:**
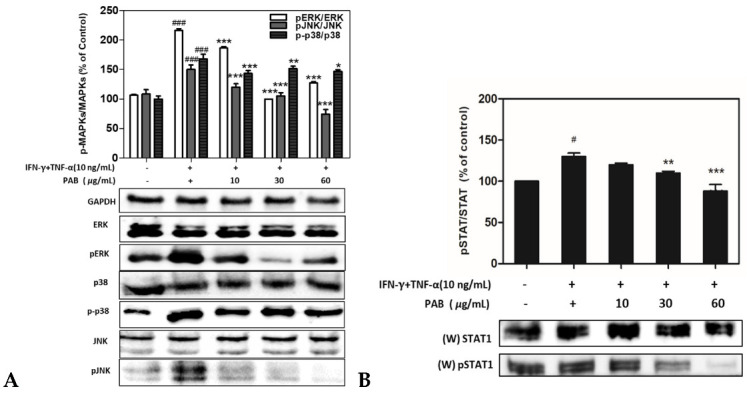
Signaling pathways of MAPK and STAT1 were measured by western-blot analysis. Cells were co-treated with varying concentrations of PAB and stimulated with IFN-γ and TNF-α (10 ng/mL each) for 24 h. Phosphorylation levels of extracellular signal-related kinase (ERK), c-Jun N-terminal kinase (JNK), and p38 are shown (**A**). The STAT1 signaling pathway was measured in whole cells (**B**). The band densities were expressed as the percentage of IFN-γ and TNF-α alone or PAB co-treated group relative to the control band by using the Image Lab analysis program. Data are presented as the mean ± SD of three independent experiments. Note: ^#^ *p*< 0.05, ^##^ *p*< 0.01, and ^###^ *p*< 0.001 versus control cells; * *p* < 0.05, ** *p* < 0.01, and *** *p* < 0.001 versus TNF-α- and IFN-γ-induced cells.

Mitogen-activated-protein-kinase (MAPK) cascades are key inflammation-signaling pathways that regulate a wide variety of cellular immune processes such as proliferation, differentiation, apoptosis, and stress responses under both normal and pathological conditions. The STAT1 pathway leads to signaling involved in the activation of p38. To determine the IFN-γ- and TNF-α-mediated MAPK and STAT1 pathways that are down-regulated by PAB in HaCaT cells, the cells were exposed to various concentrations of PAB and their expression levels were investigated using western blotting. The phosphorylation of MAPK/STAT was decreased by PAB in a dose-dependent manner (Figure 6A,B).

### 2.7. Effect of PAB on NF-κB Translocation in IFN-γ- and TNF-α-Induced HaCaT Cells

**Figure 7:**
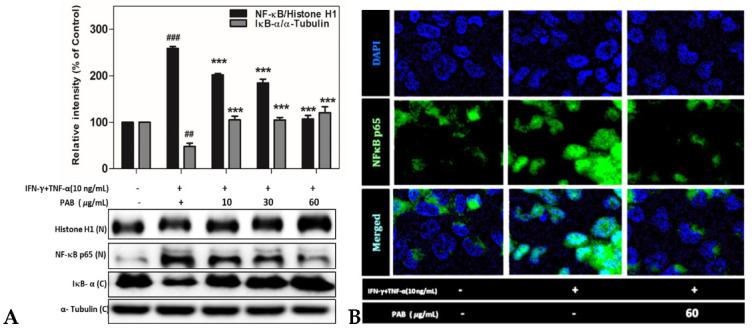
Effect of PAB on NF-κB expression in the nuclear-translocation pathway. Density of protein band was measured using western blot (**A**). Nucleus was stained with rabbit anti-NF-κB and p65, followed by Alexa Flour 1488 goat anti-rabbit IgG secondary antibody. Nuclei were identified using DAPI containing mounting medium (**B**). Data are presented as the mean ± SD of independent experiments. Note: ^#^ *p* < 0.05, ^##^ *p* < 0.01, and ^###^ *p*< 0.001 versus control cells; * *p* < 0.05, ** *p* < 0.01, and *** *p* < 0.001 versus TNF-α- and IFN-γ-induced cells.

NF-κB is regarded as a critical inducer of AD because it stimulates the transcription of chemokine genes, such as those encoding TARC/CCL17 and MDC/CCL22, as well as related genes. TNF-α and IFN-γ treatment was found to increase the transcriptional activity of NF-κB through the MAPK/STAT1 signaling pathway and to promote AD through the phosphorylation of NF-κB. Therefore, we investigated whether PAB inhibits the nuclear localization of NF-κB in TNF-α- and IFN-γ-stimulated HaCaT cells. In addition, the expression of the NF-κB protein was down-regulated by PAB (Figure 7A). The microscopy results indicated that HaCaT cells co-treated with PAB (60 μg/mL) and IFN-γ and TNF-α for 24 h showed a significant inhibition of the nuclear translocation of NF-κB (Figure 7B). These data support our hypothesis that PAB suppresses the generation of the AD-related chemokines TARC/CCL17 and MDC/CCL22 by disturbing the stimulation and nuclear translocation of NF-κB.

### 2.8. Identification of Anti-AD Compounds in PAB

**Figure 8:**
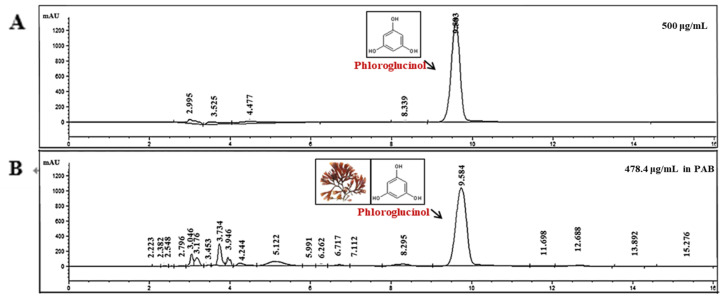
Measurement of the representative component of PAB using high-performance-liquid-chromatography analysis. The flow rate was 5.0 mL/min, and the sample-injection volume was 5 μL. Chromatogram of phloroglucinol standard concentration (Ph) of 500 μg/mL(**A**) and PAB of 10 mg/mL (**B**) was identified at a wavelength of 210 nm.

It has been consistently reported that polyphenol exists in *P. affinis* among red algae [11,21]. Based on the results of previous studies, an HPLC analysis was used to determine the effective anti-AD component of *P. affinis*. Qualitative and quantitative analyses revealed the presence of a phloroglucinol component among the polyphenols. To determine the exact amount of phloroglucinol present in PAB, a standard curve was drawn, and a quantitative analysis was performed (Figure 8). The phloroglucinol content was 478.4 μg/mL in 10 mg/mL of PAB. The phloroglucinol component of the polyphenol-based phlorotannin series was significantly detected in the butanol fraction. Phlorotannins have been reported to be abundant in brown algae, such as *Ecklonia cava* [22,23,24,25]. This study showed that the phloroglucinol component of the phlorotannin family was detected in red and brown algae. This strongly suggests that the phloroglucinol component is a mediator of the anti-AD effect of *P. affinis*.

## 3. Discussion

Keratinocytes are an important cellular model for inflammatory skin diseases, such as AD, because they produce inflammatory cytokines and chemokines. Previous studies have shown that TARC/CCL17 is highly expressed in the basal epidermis of lesioned skin in NC/Nga mice and AD patients. In addition, MDC/CCL22 levels are high in the monocyte-derived dendritic cells isolated from the venous blood and lesioned skin of AD patients. TARC/CCL17 and MDC/CCL22, which are Th2-type immune chemokines in AD, bind specifically to the CCR4 receptor and have been detected in keratinocytes. Therefore, TARC/CCL17 and MDC/CCL22 are thought to play important roles in the pathogenesis of AD. Therefore, the effect of PAB on TARC/CCL17 and MDC/CCL22 expression was investigated using TNF-α- and IFN-γ-induced human keratinocyte (HaCaT) cells. PAB significantly inhibited the protein and mRNA expressions of TARC/CCL17 and MDC/CCL22 in TNF-α- and IFN-γ-induced HaCaT cells.

In Figure 4, to investigate the effect of IFN-γ and TNF-α stimulation and PAB treatment on the gene-expression profile of HaCaT cells, the gene-expression changes in the control, IFN-γ- and TNF-α-stimulated cells, and the PAB co-treatment condition in IFN-γ- and TNF-α-stimulated HaCaT cells were analyzed using the statistical criterion *p* < 0.05. Only genes that displayed ≥ two-fold up-regulation or down-regulation were considered in this study (Figure 4A). In the IFN-γ- and TNF-α-stimulated group, the expression of 3278 genes (976 up-regulated and 1547 down-regulated) was changed; in the IFN-γ- and TNF-α-stimulated HaCaT cells of the PAB co-treatment group, the expression of 1298 genes (250 up-regulated and 293 down-regulated) was changed. Especially, the expression of inflammation-related genes such as TNFRSF9, CCL8, ICAM1, IL36G, APOL3, APOL2 [26], CXCL9 [27], and HLA-DRB1 [28] were down-regulated by PAB.

Previous studies have shown that TNFRSF9 is involved in T-cell activation and migration, and we observed that PAB suppressed the expression of the TNFRSF9 gene induced by IFN-γ and TNF-α [29,30]. When stimulated with inflammatory cytokines such as IFN-γ and TNF-α, human keratinocytes can express adhesion molecules such as ICAM-1 [31,32,33]. In some studies, the decrease in ICAM-1 expression by natural agents decreased the infiltration of immune cells into the skin, which could ameliorate inflammatory diseases of the skin [34]. Similarly, our study showed that the mRNA expression of ICAM-1 was reduced by PAB. Inflammatory chemokine levels have been shown to be elevated by induction with inflammatory stimulators such as IFN-γ and TNF-α [35]. Representative examples of inflammatory chemokines include CCL17 [36,37], CCL22 [38], CCL8 [39], and CXCL9 [40]. The gene expression of these chemokines that were induced by cytokines was suppressed by PAB. Similarly, the expression levels of IL36G, APOL3, and APOL2, which are related to inflammation, were also induced by IFN-γ and TNF-α, whereas they were suppressed by PAB. Based on previous studies, in this study, the relationship between TARC/CCL17 and MDC/CCL22 genes and nine other genes was further specified, and it was found to involve skin-inflammation-mediated genes. Hence, these genes can be used as novel AD-mediating biomarkers.

NF-κB plays a pivotal role in inducing the production of TARC/CCL17 and MDC/CCL22 [41]. Previous studies have shown that IFN-γ and TNF-α can activate NF-κB, which is responsible for the expression of pro-inflammatory chemokines and cytokines [42]. Among these cytokine stimuli, IκB proteins are phosphorylated and degraded, which allows the translocation of NF-κB into the nucleus, where it can bind to specific promoter regions of target genes and activate the expression of inflammatory-cytokine genes [43]. Thus, the inhibition of NF-κB activation plays a key anti-inflammatory role in AD. We evaluated whether the efficacy of PAB was mediated by the down-regulation of NF-κB activation. The IFN-γ- and TNF-α-induced phosphorylation of NF-κB/IκBα and degradation of IκBα were noted in the HaCaT cells treated with PAB, indicating that PAB can affect the NF-κB signaling pathway. PAB inhibited TARC/CCL17 and MDC/CCL22 expression by repressing transcriptional activation of the NF-κB promoter. These findings collectively suggest that PAB significantly inhibited the expression of TARC/CCL17 and MDC/CCL22 by blocking NF-κB activation in IFN-γ- and TNF-α-treated HaCaT cells.

STAT factors have been shown to be phosphorylated by various chemokines, and some cytokines participate in the regulation of many genes [44]. STATs are found in the cytoplasm and are phosphorylated and translocated into the nucleus upon activation. STAT1 is known to be activated by JAK or p38 MAP kinase [45,46], and upon phosphorylation, STAT1 forms homo- or heterodimers with other STATs such as STAT2 and STAT3. In this study, the phosphorylated STAT1 pathway was increased by TNF-α/IFN-γ but was inhibited in a concentration-dependent manner by PAB. The immunofluorescence assay revealed that PAB reduced the transcriptional activation of the NF-κB/IκB reporter gene as well as the reporter gene in TNF-α/IFN-γ-stimulated HaCaT cells. Western blotting showed that PAB inhibited complex formation between NF-κB and its binding-site sequence contained in the IκB mediator. The results showed that the TNF-α/IFN-γ-induced activation of NF-κB/I-κB and STAT1 was effectively attenuated by treatment with PAB in HaCaT cells.

In order to determine why these results occurred, it was confirmed what components were present in the PAB. In general, the constituents of seaweeds can be largely divided into pigment substances, amino acids, sterols, and polyphenols. Red algae are known to contain carotenoids and chlorophyll C. Previous studies have shown the UV-protection effect of *P. affinis* in HaCaT cells [47], the inhibitory effect toward allergic asthma in a murine models [11], the inflammatory effect in LPS-induced RAW 264.7 cells [48], and the skin-whitening effect in B16F10 cells [49]. However, the active ingredient in *P. affinis* has not been investigated. According to several studies, phloroglucinol-based phlorotannin compounds are known to abundantly accumulate in brown and red algae [50,51]. Phlorotannins are secondary metabolites of phloroglucinol.

Polyphenolic phloroglucinol is a bioactive substance that can be used in foods, cosmetics, and medicine. In addition, it is known to have antidiabetic [52], anti-inflammatory [53,54,55], hepato-protective [56,57], anti-plasmin inhibitory [58], and antioxidant effects [59,60]. Therefore, interestingly, this study found that phlorotannin-derived (precursor of phloroglucinol) phloroglucinol, which are mainly present in brown algae, also exist in *P. affinis*.

## 4. Materials and Methods

### 4.1. Preparation of Seaweed Materials

The dried powder of *P. affinis*, which inhabits Jeju Island, was purchased from PARAJEJU (Jeju, Korea). *P. affinis* extracts were prepared by modifying the extraction protocol described by Kang et al. [61]. The powder (2000 g) was first extracted with 80% ethanol, and fractionation was performed with a butanol layer and a water layer. The yield of primary ethanol extract was 66.9 g, and 25.82 g of the butanol fraction and 3.25 g of the water-layer fraction were obtained. Here, the water layer of *P. affinis* was expressed as PAA, and the butanol layer was expressed as PAB.

### 4.2. Cell Culture and Treatment

The HaCaT human keratinocyte cell line was purchased from the American Type Culture Collection (ATCC, Manassas, VA, USA) and cultured in DMEM supplemented with 10% fetal bovine serum (FBS; Gibco, Grand Island, NY, USA) and penicillin-streptomycin (100 U/mL; Gibco). Cells were grown at 37 °C in 100 mm dishes in a humidified atmosphere containing 5% CO_2_. The cells were passaged three times a week, and the number of passages was within 10. Briefly, HaCaT cells were seeded in 100 mm plates (1.5 × 10^6^ cells/mL) and grown for 24 h. After treatment with various concentrations of PAA (100, 500, and 1000 μg/mL) and PAB (10, 30, and 60 μg/mL) with TNF-α and IFN-γ (10 ng/mL each) or without, the cells were incubated for 24 h. The cells were collected for real-time RT-PCR and western-blot analysis. Cell supernatants were collected and used for ELISA.

### 4.3. Cell Viability

Cell viability was determined using 3-(4,5-dimethylthiazol-2yl)-2,5-diphenyl-2*H*-tetrazolium bromide (MTT; Sigma-Aldrich, St. Louis, MO, USA) assay. Briefly, HaCaT cells were plated in 96-well plates (1 × 10^5^ cells/mL) and grown for 24 h. After treatment with various concentrations of PAA (100, 500, and 1000 μg/mL) and PAB (10, 30, and 60 μg/mL) for 24 h with or without TNF-α and IFN-γ (10 ng/mL each), cells were washed with phosphate-buffered saline (PBS), treated with MTT (0.5 mg/mL), and incubated for 3 h. The insoluble formazan crystals were dissolved in 100 μL dimethyl sulfoxide (DMSO; Sigma-Aldrich St. Louis, MO, USA). After gently mixing the plate, the absorbance was measured at 570 nm using a spectrophotometer (Varioskan Lux, Thermo Fisher Scientific, Waltham, MA, USA).

### 4.4. RNA Isolation and cDNA Synthesis

Total RNA was extracted from the cells using the TRIzol reagent™ (Invitrogen, Carlsbad, CA, USA), according to the manufacturer’s instructions, and resuspended in RNase-free water. The quality of the RNA was verified at 260/280 nm using a NanoDrop^®^ spectrophotometer (DaeMyung Science, Seoul, Korea). To increase purity, RNA samples were purified using an RNeasy Mini Kit (Qiagen, NY, USA). RNA (500 ng) was used to synthesize cDNA by reverse transcription using a Toyobo cDNA kit (Toyobo, Osaka, Japan).

### 4.5. Library Preparation and Sequencing

For control and test RNAs, library construction was performed using the QuantSeq 3′ mRNA-Seq Library Prep Kit (Lexogen, Inc., Vienna, Austria) according to the manufacturer’s instructions. In brief, each 500 ng of total RNA was prepared and an oligo-dT primer containing an Illumina-compatible sequence at its 5′ end was hybridized to the RNA and reverse transcription was performed. After degradation of the RNA template, synthesis of the second strand was initiated by a random primer containing an Illumina-compatible linker sequence at its 5′ end. The double-stranded library was purified using magnetic beads to remove all the reaction components. The library was amplified to add the complete adapter sequences required for cluster generation. The finished library was purified from the PCR components. High-throughput sequencing was performed as single-end 75 sequencing using NextSeq 500 (Illumina, Inc., San Diego, CA, USA). QuantSeq 3′ mRNA-Seq reads were aligned using Bowtie2 [62]. Bowtie2 indices were either generated from the genome assembly sequence or the representative transcript sequences in order to align the genome and transcriptome. The alignment file was used for assembling transcripts, estimating their abundances, and detecting the differential expression of genes. Differentially expressed genes (DEGs) were determined based on counts from unique and multiple alignments using coverage in Bedtools [63]. The read count data were processed based on the quantile-normalization method using EdgeR within R (R development Core Team, 2016) using Bioconductor [64]. Gene classification was based on searches performed by DAVID (http://david.abcc.ncifcrf.gov/, accessed on 18 January 2022) and Medline databases (http://www.ncbi.nlm.nih.gov/, accessed on 18 January 2022).

### 4.6. Quantitative Real-Time PCR

Real-time PCR analysis was performed using an Applied CFX Connect™ real-time PCR detection system (Bio-Rad Laboratories, Hercules, CA, USA). The PCR reaction mixture was prepared using SYBR Green Real-time PCR Master Mix (Toyobo, Osaka, Japan), according to the manufacturer’s instructions. The system operates using a thermal cycler and a laser directed via fiber optics to each of the 96 sample wells. The primers used in this study are listed in Table 2. GAPDH primers were used for normalization. Thermal cycling conditions were as follows: 60 °C for 2 min and 95 °C for 10 min, followed by 40 cycles of 95 °C for 30 s, 60 °C for 30 s, and 72 °C for 30 s. The relative expression of the target genes was calculated using the CFX Connect™ Real-Time PCR detection system using the ΔΔCq method. All oligonucleotide primers were purchased from IDT Inc. (Coralville, IA, USA) and Bioneer (Seoul, Korea).

### 4.7. ELISA

ELISA was used to measure the levels of MDC/CCL22 and TARC/CCL17 in the supernatant of HaCaT cells treated with IFN-γ and TNF-α (10 ng/mL each) in the presence or absence of PAA (100, 500, and 1000 μg/mL) and PAB (10, 30, and 60 μg/mL) at the indicated concentrations for 24 h. The culture supernatant was collected and used immediately (R&D System, Minneapolis, MN, USA) according to the manufacturer’s instructions. The absorbance was measured at 450 nm using a spectrophotometer (Varioskan Lux).

### 4.8. Western Blotting

The protein concentration of each cell lysate was measured using the bicinchoninic-acid (BCA) protein assay (Bio-Rad Laboratories). Equal volumes (15 μL per lane) of each sample were loaded onto sodium-dodecyl-sulfate (SDS) polyacrylamide gels. After separation, the proteins were transferred to polyvinylidene-fluoride (PVDF) membranes (Bio-Rad Laboratories) and blocked with 2–5% bovine serum albumin (Sigma-Aldrich) or 5% skim milk in Tris-buffered saline containing 0.1% Tween-20 for 1 h [65]. The PVDF membrane, along with the primary antibodies for c-Jun N-terminal kinase (JNK1/2), phospho-JNK1/2, p38 MAPK, phospho-p38 MAPK, extracellular signal-regulated kinase (ERK1/2), phospho-ERK1/2, phospho-nuclear factor-kappa B (NF-κB), (IκBα), phospho-I kappa B kinase (IκBα), signal transducer and activator of transcription 1 (STAT1), phosphor-STAT1, Lamin b1, β-actin, and GAPDH (Cell Signaling Technology Inc., Beverly, MA, USA) were incubated at 4 °C. After washing thrice with Tris-buffered saline containing 0.1% Tween-20, the membranes were incubated with horseradish-peroxidase-conjugated secondary antibodies (anti-rabbit and anti-mouse) for 3 h. Target detection bands were observed and photographed using Chemidoc XRS (Bio-Rad Laboratories).

### 4.9. Separation of Cytosolic and Nuclear Protein Extracts

After draining the cultured medium and washing with 5 mL cold PBS, the cells were collected with an additional 3 mL of cold PBS using a scraper. The supernatant was discarded, and pellets were stored on ice. Then, 500 µL of hypotonic buffer solution was added and pipetted about 5–10 times. The cells were allowed to swell on ice for 15 min, 25 μL of detergent was added, and vortexed at high speed for 10 s. Centrifugation was performed at 14,000 rpm for 30 s. Supernatants (whole-cell extracts) were stored at −80 °C until further use. For nuclear extracts, after removing the supernatant, the pellets were resuspended in 50 μL complete lysis buffer and centrifuged at 14,000 rpm for 10 min at 4 °C, and the supernatant (nuclear fraction) was stored at −80 °C until use.

### 4.10. Immunofluorescence

HaCaT cells (1 × 10^4^ cells/slide) were co-treated with PAB, TNF-α, and IFN-γ for 1 h on a multi-chamber slide. Briefly, cells were fixed in 4% paraformaldehyde, permeabilized in 0.1% Triton X-100, and then blocked in PBS. For immunostaining, the cells were incubated with anti-NF-κB p65 antibody overnight, washed thrice in blocking buffer, and incubated with Alexa Fluor 488 anti-rabbit IgG antibody (Cell Signaling Technology Inc.) for 1 h. Immunostained NF-κB p65 was analyzed using a confocal fluorescence microscope (DMi8; Leica, Microsystems, Germany) and nuclei were counterstained with mountain solution containing 4′,6-diamidino-2-phenylindole (DAPI) (ProLong^®^ Gold with DAPI, Thermo Fisher Scientific; Life Technologies, Waltham, MA, USA, cat# P36931).

### 4.11. HPLC Analysis

To determine the representative components of PAB, high-performance-liquid-chromatography (HPLC) analysis was performed in accordance with the following method described. The HPLC system comprised an Agilent 1200 series system (Agilent Technologies, Inc. Waldbronn, Germany), UV (210 nm, 254 nm, 450 nm, 470 nm), and a column (Dikmatech, Bejing, China; 4.6 × 250 mm, 5 µm). The mobile phase used was A: 0.05% trifluoroacetic acid (TFA) in H_2_O and B: methanol. The run time was 50 min and the mobile-phase program was a gradient elution as follows: 5% (*v/v*) B at 0–20 min, 10–100% B at 20–30 min, and 5–5% B at 30.1–50 min [66]. The column oven temperature was maintained at 35 °C. Analysis was carried out at a flow rate of 0.5 mL/min with a diode array detector (DAD) at 220 nm and an injection volume of 5 μL.

### 4.12. Statistical Analysis

All statistical analyses and visualizations were conducted using GraphPad Prism 8 (GraphPad Software, San Diego, CA, USA). The results are expressed as the mean ± standard deviation (SD) or standard error of mean (SEM) of triplicate experiments. Statistical comparisons between the experimental-group and control-group values were analyzed by one-way ANOVA with Tukey’s multiple-comparison tests. * *p* < 0.05, ** *p* < 0.01, and *** *p* < 0.001 were considered statistically significant.

## 5. Conclusions

In conclusion, the pharmacological action of PAB in keratinocytes is a promising candidate for the treatment and improvement of atopic dermatitis by inhibiting the Th2 immune response. Although the exact mechanism and treatment for atopic dermatitis have not been found, controlling the excessive production of TARC/CCL17 and MDC/CCL22 may be the key to treatment. Therefore, this study proved it with the following results: (1) By down-regulating the MAPKs and STAT1 pathways with PAB, the translocation of NF-κB into the nucleus was inhibited. (2) PAB inhibited the activity of NF-κB in HaCaT cells, resulting in inhibition of TARC/CCL17- and MDC/CCL22-protein production. (3) This result was the first to investigate the existence of the phloroglucinol of polyphenols that is formed from precursors called phlorotannins, which exist in the red algae, *P. affinis*, among derived natural marine materials. (4) It is thought that the expression of additional mRNA selected by gene profiling can be utilized as a progressive result to discover new effective mechanisms and biomarkers of atopic dermatitis. Therefore, this study’s results showed that the phloroglucinol in PAB could be a potential anti-inflammation agent and a safer marine-derived, natural skin agent. This study, which proved the anti-inflammatory effect of *P. affinis*, has the advantage of being a new and progressive study in terms of “sustainable eco-friendly materials” and “recycling marine-derived materials.” Further studies revealed that phloroglucinol can be isolated directly from *P. affinis* and may contribute to its practical use. Nevertheless, in order to more accurately understand the effects of natural products, 3D cell models or artificial-skin models are needed in addition to keratinocyte cell-level experiments. Through further studies, it could be revealed that phlorotannins can be directly separated from *P. affinis* and might contribute to their practical use. Nevertheless, in addition to keratinocyte cell-level experiments, 3D cell models or artificial-skin models should be required to more accurately determine the effects of natural products.

## Figures and Tables

**Table 1:** Response of PAB on inflammatory signature genes significantly induced by IFN-γ- and TNF-α.

Inflammatory Signature Genes	IFN-γ+TNFα /Control Fold Change	PAB /IFN-γ+TNFα Fold Change	Description
TNFRSF9	13.660	0.152	tumor necrosis factor receptor superfamily member 9
MDC/CCL22	12.597	0.090	C-C motif chemokine ligand 22
CCL8	69.982	0.013	C-C motif chemokine ligand 8
ICAM1	63.309	0.045	intercellular adhesion molecule 1
IL36G	31.627	0.239	interleukin 36, gamma
APOL3	26.531	0.101	apolipoprotein L3
APOL2	10.946	0.348	apolipoprotein L2
CXCL9	3543.936	0.011	C-X-C motif chemokine ligand 9
HLA-DRB1	34.774	0.287	major histocompatibility complex, class II, DR beta 1
IDO1	1066.824	0.011	indoleamine 2,3-dioxygenase 1

Among genes related to the inflammatory response, 10 genes were selected as fold-change ≥ 10.0 and *p* value < 0.05 induced by TNF-α and IFN-γ compared to the control, and these genes were significantly inhibited by PAB.

**Table 2:** Sequences of the real-time reverse transcription polymerase chain reaction (RT-PCR) primers.

Gene	Forward (5′-3′)	Reverse (3′-5′)
GAPDH	CGT CTC CTC TGA CTT CAA CA	AGC CAA ATT CGT TGT CAT AC
MDC/CCL22	CAG CRC GAG GGA CCA ATG TG	CTT GGG GTC CGA ACA GAT GG
TARC/CCL17	ACT GCA CTC CTG GTT GTC CT	AAG GTT AGC AAC ACC ACG CC
TNFRSF9	CAG CAT GTG TGA ACA GGA TTG	GAG GAA GAA CAG CAG GAA GAG
CCL8	CTC ATG GCA GCC ACT TTC A	ATG GAA TCC CTG ACC CAT CT
ICAM1	GGC TGA CGT GTG CAG TAA TA	GGG AAA GTG CCA TCC TTT AGA
IL36G	GAA GGG CCG TCT ATC AAT CAA	CAG TCT TGG CAC GGT AGA AA
APOL3	AGG AAG ATC CAG GAG TCC ATA G	GGG TGT GAT GTC ACG CAT AA
APOL2	GAC CAA GTG AGC AGA GAG AAT C	CCA CCC ACA AAC TCC TTC AT
CXCL9	TTT CCT CTT GGG CAT CAT CTT	CTG ACC TGT TTC TCC CAC TTT
HLA-DRB1	GCT CTG TGA GTG GTT TCT ATC C	CTG AAG TCC AGA GTG TCC TTT C
IDO1	GAA ACT GGA ACT GCC TCC TAT T	GTC TTC CCA GAA CCC TTC ATA C

GAPDH, glyceraldehyde 3-phosphate dehydrogenase; MDC/CCL22, macrophage-derived chemokine; TARC/CCL17, thymus and activation-regulated chemokine; TNFRSF9, tumor necrosis factor receptor superfamily member 9; CCL8, monocyte chemotactic protein-2; ICAM1, intercellular adhesion molecule 1; IL36G, interleukin 36 gamma; APOL3, apolipoprotein L3; APOL2, apolipoprotein L2; CXCL9, chemokine (C-X-C motif) ligand 9; HLA-DRBB1, HLA class II histocompatibility antigen, DRB1 beta chain; IDO1, indoleamine 2,3-Dioxygenase 1.

## Data Availability

Not applicable.

## Sample Availability

Samples of the compounds are available from the authors.
